# What Sticks? Understanding Patient Recall in Outpatient Addiction Psychiatry

**DOI:** 10.1192/bjo.2026.11537

**Published:** 2026-06-30

**Authors:** Mohammad Ali

**Affiliations:** Addictions Recovery Community (ARC-MK), Milton Keynes, United Kingdom

## Abstract

**Aims:**

Effective outpatient addiction care depends on patients understanding and retaining clinical advice. However, the extent to which patients recall guidance after appointments is poorly studied. This evaluation aimed to assess patient recall of advice immediately after consultation and one week later, and to explore factors associated with retention.

**Methods:**

A prospective service evaluation was conducted in a UK outpatient addictions clinic. Consecutive patients attending a routine follow-up over 6 months were invited to participate (n=121). Demographics (age, gender, primary substance, housing status) and appointment characteristics (clinician continuity, consultation length) were recorded. Recall was assessed via brief structured questionnaires immediately post-consultation and at one-week follow-up, covering key treatment advice, harm-reduction strategies, and safetyguidance. Data were analysed descriptively and associations with patient or appointment factors explored.

**Results:**

Mean age was 40 years; 59% were male. Primary substances included alcohol (46%), opiates (29%), and stimulants/polysubstance use (25%). Immediate recall of all key advice was 68%, but this dropped to 42% at one week. Recall was higher among patients with continuity of care (47% vs 35%) and those attending longer consultations (>30 minutes: 52% vs <30 minutes: 38%). Housing instability and polysubstance use were associated with lower recall. Clinician confidence in patient understanding consistently exceeded actual recall.

**Conclusion:**

Patient recall of clinical advice in outpatient addiction services declines sharply within one week. Continuity of care and longer consultations are associated with improved retention. Simplifying messages, repeating key advice, and providing written or digital reinforcement may enhance understanding and engagement, with implications for treatment planning and service effectiveness.

